# Communications enhance sustainable intentions despite other ongoing crises

**DOI:** 10.1007/s11625-024-01556-9

**Published:** 2024-09-14

**Authors:** Ngoc T. H. Nguyen, Simon Willcock, Louise M. Hassan

**Affiliations:** 1 Bangor Business School, Bangor University, Bangor, UK; 2 Lincoln International Business School, University of Lincoln, Lincoln, UK; 3 School of Tourism, University of Economics Ho Chi Minh City, Ho Chi Minh City, Vietnam; 4 School of Environmental and Natural Sciences, Bangor University, Bangor, UK; 5 Net-Zero and Resilient Farming, Rothamsted Research, Harpenden, UK; 6 Birmingham Business School, University of Birmingham, Birmingham, UK

**Keywords:** Behavior change, COVID-19, Crisis, Mask wearing, Sustainability, Trade-off

## Abstract

There is an ongoing trend toward more frequent and multiple crises. While there is a clear need for behaviors to become more sustainable to address the climate crisis, how to achieve this against the backdrop of other crises is unknown. Using a sample of 18,805 participants from the UK, we performed a survey experiment to investigate if communication messages provide a useful tool in nudging intentions toward improved sustainability in the context of the COVID-19 pandemic. We found that, despite the ongoing COVID-19 crisis, media messaging resulted in increases in sustainability-related intentions for all our communication messaging conditions. Specifically, after our communication was presented, (i) almost 80% of people who were not currently recycling their surgical masks reported their intention to do so; there was a > 70% increase in both (ii) the number of people likely to pick up face mask litter and (iii) the number of people willing to disinfect and reuse their filtering facepiece (FFP) masks 4–6 times, while (iv) there was an increase by 165% in those who would wash cloth masks at 60 °C. Our results highlight that communication messaging can play a useful role in minimizing the trade-offs between multiple crises, as well as maximizing any synergies. To support this, decision-makers and practitioners should encourage the delivery of sustainability advice via multiple sources and across different types of media, while taking steps to address potential misinformation.

**Supplementary Information:**

The online version contains supplementary material available at 10.1007/s11625-024-01556-9.

## Introduction

The COVID-19 pandemic was an unprecedented global crisis. The first confirmed case was recorded in December 2019 and then the number of confirmed cases substantially increased to above 7800 cases worldwide in late January 2020 (World Health Organization 2020b). The official death toll from COVID-19 surpassed one million by late September 2020 (Ioannidis 2020). To slow the rate of transmission of the virus and protect public health, health professionals encouraged the practice of wearing face masks (World Health Organization 2020a). Many governments introduced national guidance on the use of mask coverings and instigated their use in workplaces and public buildings (Roberts et al. 2022).

However, decision-makers and citizens were unable to focus on this health crisis alone as there were (and are) multiple ‘wicked’ problems that require urgent attention. For example, across the globe, we observe widespread climate change (IPCC 2023), biodiversity loss (Hill et al. 2018), and a global decline in the benefits people receive from nature (IPBES 2019; Ruckelshaus et al. 2020). Importantly, the consequences of unsustainable practices in response to COVID-19 may have contributed to the severity of these crises (Prata et al. 2020; De-la-Torre and Aragaw 2021; Roberts et al. 2022). Although wearing masks may have contributed to a reduction of the COVID-19 outbreak (World Health Organization 2020a), the use of face masks adds another stress on the environment (Klemeš et al. 2020; Prata et al. 2020; Roberts et al. 2022). For example, surgical masks are mainly made of non-biodegradable plastics and can take 450 years to break down (Dybas 2021). A single surgical face mask can release as many as 173,000 microfibers per day into the seas, which is damaging to marine life (Saliu et al. 2021). In 2022, surveys indicated that face masks accounted for more than 5% of all litter in the UK (Roberts et al. 2022).

Thus, there is a clear need to act in a more sustainable way even when faced with other ongoing crises—particularly as there is an ongoing trend toward more frequent and multiple crises, ranging from the climate crisis to a cost of living crisis to the COVID-19 pandemic (Gear 2022; Pinkwart et al. 2022). While there is a clear need for behaviors to change to become more sustainable, is it possible to change behaviors given a multiple crisis backdrop or are people too busy ‘fighting the fires’ of multiple crises to make substantial change? And, if it is possible, how can this behavior change be stimulated? While people report positive attitudes toward sustainable consumption (Trudel and Cotte 2009), they often hesitate to act sustainably (Devezer et al. 2014). This is partially because the payoff of acting unsustainably is certain (i.e., immediate gratification in the here and now), whereas some of the favorable outcomes of sustainable consumption will benefit the environment, the society and the economy external to the self (White et al. 2019) and may be only seen in the distant future (Amel et al. 2017).

Media messaging can influence public opinion on social issues, as shown in communication theories and models, such as agenda setting ideas (McCombs and Shaw 1972). Media agenda building refers to the attempts that individuals/organizations/institutions have on how people perceive the objects of the communication messaging or convey the agenda of what is crucial to someone (McQuail 2010). During the COVID-19 crisis, many studies focused on the effect media has on changing perceptions about health-related risks and how to conduct health protection practices properly (e.g., Lee and Li 2021; Romer and Jamieson 2021; Liu et al. 2022). Importantly, media communication builds trust even when presented with uncertainties. For example, as the public health crisis evolved (e.g., H1N1 influenza), uncertainties were high, but health warnings may still have impacted public health behaviors (Bish and Michie 2010). However, there have been inconsistences surrounding the impact of such communications. For example, Romer and Jamieson (2021) and Liu et al. (2022) both carried out investigations starting in the first half of 2020 (i.e., during COVID-19 pandemic). Romer and Jamieson (2021) found no significant impact of media messaging on mask-wearing behaviors, whereas Liu et al. (2022) highlighted its positive effect on intentions to wear masks.

The top-down framework (also called the deficit model; Durant 1995) has been well documented for health and policy communications for many decades (Porat et al. 2020). It refers to media agenda settings starting from ‘the science or evidence’. This framing prioritizes the accuracy and the importance of the message derived from science which is essential to inform policies and to fill in public knowledge gaps by experts’ advisories. The top-down approach was deemed suitable due to the unprecedented nature of the COVID-19 pandemic and the limited awareness among the audience regarding the environmental implications of health practices related to COVID-19 (i.e., knowledge gap). The messaging used in this study was supported by scientific evidence, including guidance on FFP (filtering facepiece) mask disinfection methods (evidence in the message supported by Ludwig-Begall et al. 2021) and the recommended washing temperature for cloth masks (NHS England and NHS Improvement 2020; and Brennan et al. 2021; Table 1). Employing this top-down strategy significantly bolstered the credibility of the messaging and helped address knowledge deficiencies among the audience, thereby encouraging their engagement in sustainable practices amidst uncertain circumstances.

**Table 1:** A summary of the dependent variables and analysis strategies for each communication messaging condition used in our survey experiments

Communication messaging conditions	Communication message	Before the communication message	After the communication message	Analysis strategy
Picking up face mask litter (evidence in the message supported by Roberts et al. 2022)	The most recent statistics indicate that face masks account for more than 5% of all litter in the UK Take part in your local face covering litter pickup on Saturday 10th September! Using gloves and litter pickers, you can safely pick up litter face masks and put them in general waste bins	How likely are you to pick up face covering litter you see in public places? (1—Extremely unlikely to 7—Extremely likely	Based on this information, how likely are you to take part in your local face covering litter pickup? (1—Extremely unlikely to 7—Extremely likely)	A repeated-measures ANOVA analysis to determine if the likelihood of picking up face covering litter differed before versus after the messaging was presented, and if this difference in consumer behavior was affected by the types and sources of media
Recycling surgical masks (evidence in the message supported by Dybas 2021, Saberian et al. 2021 and Saliu et al. 2021)	6.8 billion surgical mask are used across the world each day Surgical masks are mainly made of non-biodegradable plastics and can take 450 years to break down. A single surgical face mask can release as many as 173,000 microfibers per day into the seas, which is damaging to marine life Single-use surgical face coverings can be disposed of in TAPAT stores across the country as part of a recycling scheme in partnership with Reworked and Scan2Recycle	Do you currently recycle your surgical face covering? (0—No, 1—Sometimes, 2—Always)	Based on this information, how likely are you to recycle your surgical face covering? (1—Extremely unlikely to 7—Extremely likely)	One-sample *t* tests were conducted to examine if the likelihood of recycling surgical masks is higher than the mid-scale after presenting the message An ANOVA analysis was used to examine if the likelihood of adopting the advisory was explained by previous behaviors and the media. This analysis included consumers’ previous behaviors before viewing the information, media types, media sources and their two-way and three-way interactions along with controlling for gender and age
Disinfecting FFP masks (evidence in the message supported by Ludwig-Begall et al. 2021)	FFP-type masks should not be worn on consecutive days. Coronavirus decreases significantly in infectiousness if a mask is left to dry at room temperature for seven days For example, you could have seven hooks for the seven days of the week, and once you have worn Monday’s mask, for example, you can replace it on the Monday mask hook, and on Tuesday wear your Tuesday mask. The following Monday, if there was any coronavirus on your face covering, the pathogen will no longer be infectious and you can wear the face covering again If you are wearing on FFP face covering per day, you can hang the mask on the hook in a dry space that has sufficient room for seven masks to hang next to each other. This procedure cannot be undertaken in bathrooms or kitchens due to the increase in humidity and should not be undertaken outside either as it is too cold to reduce the pathogen The drying process can be repeated five times, and then you can use your FFP mask one last time and discard it, so each mask should be worn a maximum of 6 times in total You should only wear FFP masks for two hours at a time, so it might be best to wear this type of face covering when in high-risk situations, such as when traveling on public transport or when visiting a medical center. You should not use this procedure for masks that have been directly coughed on, that have come into contact with an infected person or if the face covering is particularly damaged in any way	Please indicate how many times you wear your FFP face covering before disposing of it (1, only once; 2, 2–3 times; 3, 4–6 times; 4, 7–9 times; 5, 10 or more times)	Given this information about disinfecting your face covering, please indicate how many times you will wear your FFP-type face covering in future before disposing of it (1, only once; 2, 2–3 times; 3, 4–6 times; 4, 7–9 times; 5, 10 or more times)	A descriptive analysis for frequency of disinfecting FFP masks before and after viewing the advisory A PROBIT regression was used to determine if the media and past behavior explained the frequency of disinfecting an FFP mask after viewing the message The model included the frequency that people previously wore an FFP mask before viewing the information, media types, media sources and their two-way and three-way interactions along with controlling for gender and age
Washing cloth face coverings (evidence in the message supported by NHS England and NHS Improvement 2020; and Brennan et al. 2021)	A cloth face covering should be washed in a washing machine daily at the hottest possible temperature— ideally at 60^0^C or above with your standard washing liquid or powder Washing at higher temperatures kills any coronavirus on the face covering, making your face covering safe to wear again	What temperature do you wash your cloth face covering at (1–30 °C; 2–40 °C; 3–50 °C; 4–60 °C; 5–70 °C; 6–80 °C; 7–90 °C)?	Based on this information, what temperature will you wash your cloth face covering at (1–30 °C; 2–40 °C; 3–50 °C; 4–60 °C; 5–70 °C; 6–80 °C; 7–90 °C)?	A descriptive analysis for cloth mask wearers’ choice of washing temperature A PROBIT regression was used to determine if the media and past behavior of washing temperature explained the current choice of washing temperature after the message was presented The model included past behavior of washing temperature before viewing the information, media types, media sources and their two-way and three-way interactions along with controlling for gender and age

During a crisis, it is not only the message being communicated that is important, but it also matters ‘who’ oversees communication (e.g., local government and health institutes) (Larson and Heymann 2010; Quinn et al. 2013). For example, most of the messages investigated in previous studies during public health crises or the COVID-19 pandemic involved communications by state governments and health institutes (e.g., Quinn et al. 2013; Lee and Li 2021; Romer and Jamieson 2021). During health emergencies, government and public health professionals need to communicate effectively to enhance public resilience (Rubin et al. 2009; Vardavas et al. 2021) and encourage risk-reducing behaviors (e.g., vaccinations; Bish et al. 2011). Similarly to when patients look for guidelines and feedback from practitioners, the audience relies on the health communication from the government and health institutes due to trust building (Porat et al. 2020).

In some contexts where trust building between the government and citizens is problematic (Parsons and Wiggins 2022), people are more likely to be reliant on advisories from other parties. A census report in 2023 shows only 1 in 5 adults in Great Britain indicated their trust in the UK government (Office for National Statistics 2024). Research on the role of other parties (e.g., celebrities, companies) has received limited attention, with notable exceptions. For example, celebrity spokesperson Tom Hanks achieved the same level of respondents’ willingness to re-share a call to social distancing as the Government did (Abu-Akel et al. 2021).

Liu et al. (2022) showed that mass or mainstream media (e.g., newspapers, TV) was more effective at changing intentions to wear masks than social media, because social media includes user-generated content with little scrutiny and so is perceived as lacking in credibility. However, social media is considered as a common communication means; for example, Twitter is one of the prominent social networking sites and has over 330 million active users sending around 6000 status updates, or tweets, every second globally (Turner 2024). During the COVID-19 pandemic, social media has been widely used as an essential communication means by governments, organizations and educational institutions (Gao et al. 2020). Social media becomes relevant in this context because it enables real-time and two-way interactive communication, knowledge exchange, information sharing and trust building (Lovejoy and Saxton 2012; Saffer et al. 2013). Health organizations, therefore, use Twitter as a popular platform for health promotion and public participation (Park et al. 2016) as well as for understanding public perceptions/misconceptions and their information needs about COVID-19 (Hauer and Sood 2020).

**Fig. 1:**
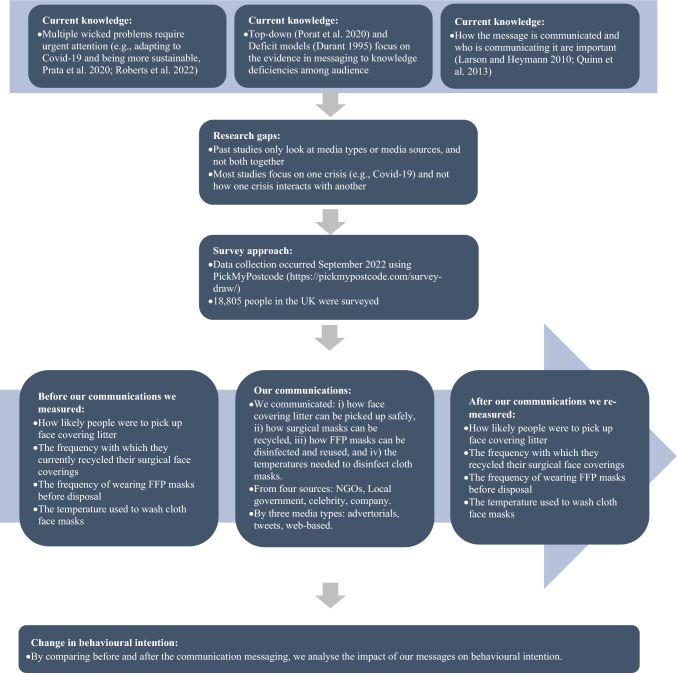
The motivation and methodological approach of our study

Research on communication messaging about sustainable practices under the backdrop of a public health crisis has remained limited (e.g., Ayman et al. 2020). While previous research has paid due attention to the role of either media types or media sources, this study expands previous investigations by studying both multiple media types (i.e., tweets, advertorials, web-based news) and media sources (i.e., local government, non-governmental organizations [NGOs], companies, celebrities) across multiple behavioral contexts related to mask wearing (Fig. 1). We particularly focus on enhancing more sustainable practices (i.e., picking face mask litter, adopting FFP disinfection measures, recycling surgical masks and washing cloth masks at the lowest safe temperature), providing useful insights into how more sustainable behaviors can be encouraged against a backdrop of other crises. We did so using a survey experiment run with 18,805 people across the United Kingdom (UK) in the context of the COVID-19 crisis in September 2022.

## Materials and methods

### Data collection

Data collection occurred in September 2022. The surveys were distributed using Pick My Postcode—a free, postcode lottery website through which people can complete surveys (https://pickmypostcode.com/survey-draw/). With every survey, completed members build a cash bonus, which they have a chance to win alongside the prize money that is awarded to winners randomly drawn from the postcodes. We targeted all postcodes across the UK. Individuals signed up to Pick My Postcode were notified on the survey page that there was a survey available for their postcode with a bonus of £1. While Pick My Postcode recruited the participants, the survey was developed and completed on the Qualtrics platform.

The aim of our survey was to conduct an experiment to determine if sustainable behaviors related to the use of face masks were likely to be changed by communication messaging delivered from varied types and sources of media (SI-1). We manipulated the communication messaging delivered across three types of media (tweets vs. web-based news vs. advertorials) and four sources of media (company vs. NGO vs. celebrity vs. local government; Fig. 1). We used the fictional, neutral company name TAPAT (e.g., Johnson et al. 2011), but for the other treatments (NGO vs. celebrity vs. local government) we used real entities—Carbon Trust for the NGO condition, and Nottinghamshire County Council for the local government condition. Participants were to assume they were from the local County Council that was used in the experiment (‘*Imagine that you are a resident within the Nottinghamshire County Council area*’). The celebrity was a widely respected environmentalist within the UK, whose name is not included here for confidentiality reasons.

Participants were first asked if they wore face masks during the COVID-19 pandemic and if they chose either FFP masks or surgical masks or cloth face coverings. Our experiment then collected participants’ responses to more sustainable behaviors before and after the communication messaging was presented to participants (Fig. 1). Specifically:

- Those who did not wear masks were led to the *picking up face mask litter* condition. Their intention to pick up face mask litter was observed before versus after reading the communication message that introduced a local campaign to pick up face covering litter along with instructions for safely partaking in the activity (SI-1).
- Those who wore surgical masks were allocated into the *recycling surgical masks* condition (SI-1). They were asked the frequency with which they currently recycled their surgical face coverings. Subsequently, their intention to recycle their surgical masks was captured on a 7-point scale (1–*Extremely unlikely* to 7—*Extremely likely*) after reading the manipulated message that specified the negative impact of discharging surgical masks into the environment and then introduced a recycling scheme at TAPAT stores. When reading the message, participants first received a preamble that referred to a well-known brand that had operated a mask recycling scheme that for confidentiality purposes was being disguised as “TAPAT”. TAPAT is fictional and was described as a supermarket that was widely available in the UK (SI-1).
- Those who wore FFP masks were assigned to the condition of *disinfecting FFP masks*. The message indicated that FFP mask wearers could disinfect their FFP masks and do so a maximum of five times (SI-1). Participants were asked their frequency of wearing their FFP mask before disposing of it by a multiple-choice question. Behavior regarding re-wearing of an FFP mask was asked twice—once before viewing the message (“*Please indicate how many times you wear your FFP face covering for before disposing of it*”) and once after presenting the message (“*Given this information about disinfecting your face covering, please indicate how many times you will wear your FFP type face covering in future before disposing of it*”).
- Those who wore cloth face coverings were assigned into the *washing cloth face coverings* condition. The message provided the recommendation that cloth masks should be washed at 60 °C or above to ensure safe use (SI-1). The participants were asked about their choice of temperature to wash their cloth mask before viewing the message (“*What temperature do you wash your cloth face covering at?*”) and after the message was presented (“*Based on this information, what temperature will you wash your cloth face covering at?*”).

### Data analysis

Descriptive statistics show the demographic information of the respondents along with their behaviors of wearing masks during the COVID-19 pandemic. A sample of 18,805 participants was recruited for the research (*M*_age_ = 53.43, 63% female; SI-2). Most participants were from England (84%). Nearly half of the sample reported that they wore masks in the past month (8153 obs.), of which the majority of mask wearers used surgical masks (3798 obs.), followed by cloth masks (2961 obs.) and the FFP type (1379 obs.). Within the communication messaging conditions (described above), our samples were randomly and equally assigned to four conditions of media sources (company vs. NGO vs. celebrity vs. local government) and three conditions of media types (tweets vs. web-based news vs. advertorials; SI-2).

The overall aim of this research was to determine if sustainable behavior change was associated with different media types and sources of communication messaging. Descriptive statistics and* t* tests were applied to analyze whether or not consumers’ behavioral intentions related to mask wearing were changed by the communication messaging. In addition, regression tests were applied to assess the relationships between mask wearing behaviors with media communication types and sources. Sustainable intentions were captured by varied levels of measurement in different conditions of mask wearing behaviors, and Table 1 summarizes analysis techniques that were employed across four conditions of communication messaging. To evaluate the behavioral change resulting from the communication messaging in the condition of picking up face mask litters, a repeated-measures ANOVA was the primary analysis method, in which intentions to pick up face mask litters before versus after the messaging was the dependent variable, and independent variables include types of media and sources of media. In the condition of recycling surgical masks, three-way interaction ANOVA was primarily employed to examine the interaction effect of past recycling behaviors, media types and media sources on the recycling behavioral intentions after reading the messaging. In the conditions of disinfecting FFP masks and washing cloth masks, a PROBIT regression was applied to analyze the three-way interaction of past behaviors (FFP disinfection/washing temperature selection for cloth masks), media types and media sources on the behavioral intentions after reading the related messaging.

Sankey diagrams were employed to visually map the flow of changes that existed in consumer behaviors before versus after the communication messaging was presented. All analyses were conducted using SPSS (Version 27, George and Mallery 2021) and STATA (Version 12, Hamilton 2012) packages.

These analyses show that, despite the ongoing COVID-19 crisis, media messaging results in increases in sustainability-related intentions. A potential societal benefit of this research is that, during future crises, communication messaging could be better applied to ensure sustainability goals are maintained.

## Results

Despite the ongoing COVID-19 crisis, we found that communication messaging resulted in increases in sustainability-related intentions for all our communication messaging conditions (Table 2). Here, we present the results from each one of the four conditions in turn. The data are freely available via https://reshare.ukdataservice.ac.uk/856661/.

**Table 2:** A summary of the impact of each communication messaging condition on sustainability-related intentions of our 18,805 participants, highlighting the overall outcome of the messaging intervention (i.e., captured by the adopted intention), the magnitude and significance of the change compared to stated intentions prior to the messaging intervention, as well as any significant interaction terms

Communication messaging conditions	Overall outcome of the messaging intervention (to adopt the advisory)	Drivers of the messaging intervention			
		Effect of past behavior	Effect of media types * past behavior	Effect of media sources * past behavior	Effect of media types * media sources* past behavior
Picking up face mask litter	*M* = 2.99, *t* = – 52.81, df = 10,651, *p* < 0.001, significantly lower than the midpoint of the scale (4) (*1—Very unlikely to 7—Very likely*)	Repeated measure ANOVA: *F*(1,10,638) = 15.01, *p* < 0.001	Repeated measure ANOVA: no interaction effect found	Repeated measure ANOVA: *F*(3,10,638) = 11.00, *p* < 0.001	Repeated measure ANOVA: no interaction effect found
Recycling surgical masks	*M* = 5.61, *t* = 53.09, df = 3797, *p* < 0.001, significantly higher than the midpoint of the scale (4) (*1—Very unlikely to 7— Very likely*)	Univariate ANOVA: *F*(2,3760) = 157.29, *p* < 0.001	Univariate ANOVA: no interaction effect found	Univariate ANOVA: no interaction effect found	Univariate ANOVA: no interaction effect found
Disinfecting FFP masks to maximum 5 times	*N*_4–6times_ = 476 (34.5%); *N*_2–3times_ = 357 (25.9%); *N*_once_ = 337 (24.4%)	PROBIT regression: *b* = 1.05, *z* = 6.71, *p* < 0.001	PROBIT regression: *b* = – 0.16, *z* = – 2.19, *p* = 0.03	PROBIT regression: no interaction effect found	PROBIT regression: no interaction effect found
Washing cloth face coverings at the recommended 60 °C temperature	*N*_60ºC_ = 1499 (56.50%); *N*_below 60ºC_ = 851 (32.08%); *N*_above 60ºC_ = 215 (8.10%)	PROBIT regression: *b* = 0.40, *z* = 4.69, *p* < 0.001	PROBIT regression: no interaction effect found	PROBIT regression: no interaction effect found	PROBIT regression: no interaction effect found

### Picking up face mask litter

**Fig. 2:**
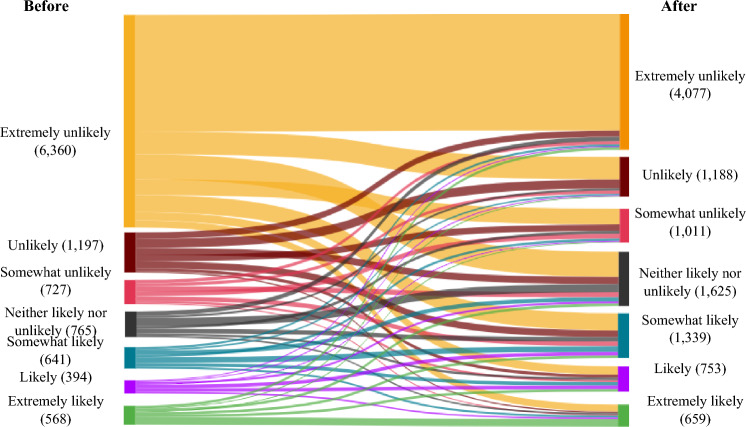
The change in intentions to pick up face mask litter before and after viewing the messaging (*N* = 10,652). Participants were asked to indicate the degree to which they were likely to pick up face mask litter before and after viewing the messaging on a seven-point scale (from 1—Extremely unlikely to 7—Extremely likely)

A repeated-measures ANOVA analysis was conducted, in which the dependent variable was the change in individuals’ intention to pick up mask litter prior to versus after media messages were presented as well as independent variables which were media types and media sources. The result found that the likelihood that people picked up mask litter was significantly greater after reading the communication message than before (*M*_before_ = 2.21, *M*_after_ = 2.99, *F*(1,10,638) = 15.01, *p* < 0.001; summarized in Table 2, with more detail in SI-2). The number of people who reported ‘*extremely unlikely*’ to pick up face mask litter decreased by 35.90% (*N*_before_ = 6360, *N*_after_ = 4077; Fig. 2). The number of people who reported from ‘*Somewhat likely*’, ‘*Likely*’ to ‘*Extremely likely*’ to pick up face mask litter increased by 71.62% (*N*_before_ = 1603, *N*_after_ = 2751). Importantly, the difference in intentions to pick up mask litter before versus after viewing the message was statistically significant across media sources (*F*(3,10,638) = 11.00, *p* < 0.001; Table 2). People were more likely to adopt the advisory and engage in picking up litter if the source was the local council, compared to other media sources (e.g., NGO, celebrity and company; see SI-2-2 for more detail). However, there was no significant difference in intentions to pick up mask litter before versus after the message was presented between groups of media types (*F*(2,10,638) = 2.32, *p* = 0.11), or groups of both media types and media sources (*F*(6,10,638) = 1.42, *p* = 0.20; Table 2).

Despite the communication message having a positive impact on intention to engage in picking up face mask litter, the change in their intention to adopt this advisory was low. A one-sample *t* test found that the average of intentions to pick up face mask litter were recorded to be significantly less than the midpoint of the scale (4; scale ranging from 1—*Extremely unlikely* to 7—*Extremely likely*) both before and after the communication messaging (*M*_before_ = 2.21, *t* = – 100.56, df = 10,651, *p* < 0.001, *M*_after_ = 2.99, *t* = – 52.81, df = 10,651, *p* < 0.001; Table 2 and Fig. 2). People were unlikely to pick up face covering litter because this course of action was considered unsafe (34%), they were not available to engage in the activity (33%) or they did not perceive it to be their responsibility to do so (31%; SI-2). Additionally, their low interest in the activity (23%) and health-related issues (18%) are barriers to perform this behavior.

### Recycling surgical masks

**Fig. 3:**
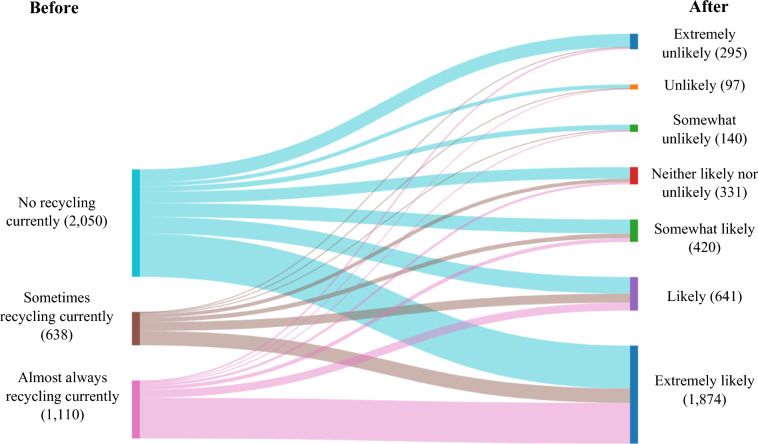
Change in intentions to recycle surgical masks before and after viewing the messaging (*N* = 3798). Before viewing the message, people answered (1) No recycling currently, (2) Sometimes recycling currently, (3) Almost always recycling currently. After viewing the message, people were asked to indicate the degree to which they were likely to recycle their surgical mask on a seven-point scale (from 1—Extremely unlikely to 7—Extremely likely)

Among nearly 3800 participants who wore surgical masks in the past month, more than half (54%) of them reported that they were not currently recycling their surgical masks (Sometimes: 16.8%; Always: 29.2%; Fig. 3). However, the communication messaging changed their intention regarding recycling behavior. A one-sample *t* test analysis showed the likelihood that these participants recycled their surgical masks after the communication messaging were significantly higher than the mid-scale (1—*Extremely unlikely* to 7—*Extremely likely*; *M* = 5.61, *t* = 53.09, df = 3797, *p* < 0.001; Table 2). After viewing the message 49.3% of participants reported ‘*Extremely likely*’ to recycle masks in future. A univariate ANOVA test showed that the intentions to recycle surgical masks were significantly influenced by past behavior (i.e., whether they previously recycled their surgical masks; *F*(2,3760) = 157.29, *p* < 0.001; summarized in Table 2, with more detail in SI-2). Almost 80% of consumers who were not currently recycling their surgical masks reported a higher likelihood to recycle their masks after viewing the message (Fig. 3). However, the interaction of past behavior, media types, and sources (*F*(12,3760) = 0.556, *p* = 0.88), that of media sources and past behavior (*F*(6,3760) = 3.88, *p* = 0.29) and that of media types and past behavior (*F*(4,3760) = 3.74, *p* = 0.32) did not reach statistical significance (Table 2). A one-sample *t* test found that those who reported that they did not recycle their surgical mask previously rated significantly higher likelihood than the scale midpoint (4) of the 1-to-7 point scale (*N* = 2050, *M* = 5.15, df = 2049, *p* < 0.001); same for those who reported ‘S*ometimes’* recycling (*N* = 638, *M* = 5.84, df = 637, *p* < 0.001); and reported ‘A*lways’* recycling (*N* = 1110, *M* = 6.32, df = 1109, *p* < 0.001). Surgical mask wearers were less likely to recycle their masks because there was no mask recycling point/bin near them (78%) or because a recycling box was not affordable (27%; SI-2). Too much effort (12%) was also one of the important barriers of surgical mask wearers to recycle masks.

### Disinfecting FFP masks

**Fig. 4:**
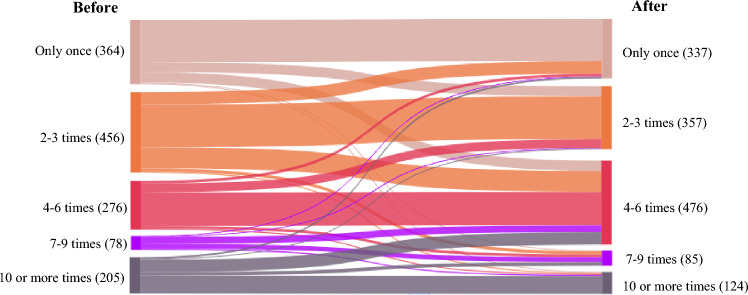
Change in intentions to disinfect FFP masks before and after viewing the messaging (*N* = 1379). Participants were asked to report the number of times they would disinfect their FFP masks before viewing the messaging and the times that they would disinfect their FFP masks after viewing the messaging (i.e., only once; 2–3 times; 4–6 times; 7–9 times; 10 or more times)

Advising about disinfecting FFP masks (to a maximum of five times) was effective in persuading FFP mask wearers to use their masks more times before disposing of it (Fig. 4). In line with the advice, 34.5% of people (*N* = 476) adopted the intention to disinfect their FFP masks and wear this mask “*4–6 times*” after providing the information, an increase from 20% (*N* = 276) before (Fig. 4). Similarly, after viewing the information, the number of participants intending to wear FFP masks “*2–3 times*” reduced by 38.4% (*N*_difference_ = 99) and those intending to wear them “*only once*” reduced by 8.7% (*N*_difference_ = 27). Results of the PROBIT regression found the likelihood that participants who adopted the advice was determined by past behavior (i.e., whether they previously reused masks; *b* = 1.05, *p* < 0.001) and the interaction between past behavior and media types (*b* = – 0.16, *p* = 0.03; summarized in Table 2, with more detail in SI-2). However, the interaction of past behavior, media types and sources (*b* = 0.05, *p* = 0.06) and that of media sources and past behavior (*b* = – 0.10, *p* = 0.07) did not significantly affect the number of times FFP masks would be worn after the communication messaging was presented (Table 2). Skepticism about the effectiveness of the disinfection measure in the messaging was the main reason as to why FFP mask wearers did not disinfect their masks (43% of the FFP mask wearers) (SI-2). The disinfection method was also found difficult (12%) to adopt.

### Washing cloth face coverings

**Fig. 5:**
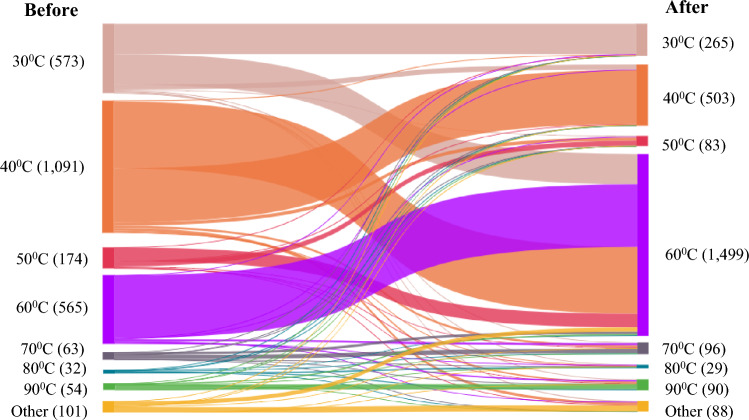
Change in intentions to choose temperature to wash cloth face coverings before and after viewing the messaging (*N* = 2653, with 308 missing values being excluded). Participants were asked to report the current temperature of washing their cloth face coverings before viewing the messaging and the temperature that they would wash their cloth face coverings at after viewing the messaging (i.e., 30 °C, 40 °C, 50 °C, 60 °C, 70 °C, 80 °C, 90 °C, other)

After viewing our advisory that cloth masks should be washed at 60 °C or above, many cloth mask wearers changed their intention and indicated that they now intended to wash their mask at 60 °C—an increase of 165% (*N*_before_ = 565, *N*_after_ = 1499; Fig. 5). The number of participants intending to wash their cloth masks above 60 °C increased by 44.30% (*N*_difference_ = 66), but fewer participants intended to wash their cloth mask at 30 °C (*N*_difference_ = 308, 53.75% change), 40 °C (*N*_difference_ = 603, 53.90% change) and 50 °C (*N*_difference_ = 89, 52.30% change; Fig. 5). Results of the PROBIT regression found the significant effect of past behavior (i.e., choices of washing temperature prior to the messaging) in predicting the temperature that people washed their cloth mask after viewing the messaging (*b* = 0.40, *p* < 0.001; summarized in Table 2, with more detail in SI-2). However, the interaction of past behavior, media types and sources (*b* = 0.004, *p* = 0.80), that of media sources and past behavior (*b* = 0.002, *p* = 0.94) and that of media types and past behavior (*b* = – 0.005, *p* = 0.90) did not significantly affect the choice of washing temperature that people adopted after viewing the advisory (Table 2). The top three reasons as to why people did not wash their cloth mask at 60 °C included “*I do not do anything at 60* °*C*” (55%), “*I don’t trust that washing at recommended temperature kills any coronavirus and makes the face mask safe to use again*” (11%) and ‘*It is too much effort*’ (7%; SI-2).

## Discussion and conclusions

In agreement with previous research, we found communication messaging was effective in changing intentions toward sustainability-related behaviors (e.g., Shahzalal and Hassan 2019; Son et al. 2022). However, importantly, we show that this positive impact is robust to the presence of another ongoing crisis—in our case, the COVID-19 pandemic (Table 2). There has been some debate as to whether sustainability goals can be achieved in the face of other crises, or whether attempts to solve one crisis trades-off against another (e.g., the increase in use of single-use masks and associated litter in an attempt to minimize the impact of COVID-19; Mikulčić et al. 2021; Elsamadony et al. 2022). Across all communication messaging conditions, we show that, despite the COVID-19 crisis, intentions can be nudged toward sustainability. For example, our results show that messaging was useful in increasing intentions to: (a) engage in picking up face mask litter, (b) recycle surgical masks, (c) safely reuse FFP masks, and (d) adopt washing of cloth masks at the lowest safe temperature (Table 2; Figs. 2, 3, 4, 5).

Our results do not indicate a lack of trade-offs across crises. We show that, for example, by changing intention to predominantly wash cloth masks at 60 °C, many participants were increasing the temperature of their wash (i.e., from 30 to 40 °C; Fig. 5), requiring more energy and thus impacting sustainability goals. However, others that were previously washing cloth face masks at temperatures higher than 60 °C—presumably to ensure the masks were safe to re-wear—show intentions to reduce the temperature of their washes, achieving a win–win in regard to COVID-19 and sustainability (Fig. 5). Similarly, while we observed significant increases in intentions to pick up face mask litter (Table 2), the change in intention was small. Nevertheless, given the huge increase in use of masks during the pandemic due to COVID-19 legislation (Prata et al. 2020), the net-effect was a large increase in litter (Roberts et al. 2021) and a net-negative impact on sustainability goals (Elsamadony et al. 2022). As such, even a small improvement in picking up litter would have sustainability benefits. Indeed, from August to October 2020 the UK had a higher overall proportion of litter from masks, gloves and wipes than in some EU countries, Australia and the USA (Roberts et al. 2022). Thus, our results suggest trade-offs between multiple crises (Elsamadony et al. 2022), but that communication messaging can play a useful role in minimizing these trade-offs, as well as maximizing any synergies.

We found that the impact of specific types and sources of media varied across communication messaging conditions. For example, media sources (i.e., local government, NGO, company and celebrity) played a role in changing intentions to pick up face mask litter. In this case, the local government was found to be the most effective source of media to enhance the likelihood that people might engage in picking up face mask litter (SI-2). This result is in line with previous studies that show that, as trusted sources, local governments have a large sway over behavioral change (Quinn et al. 2013; Lee and Li 2021). By contrast, tweets and advertorials were found to be most effective in increasing intentions to disinfect and reuse FFP masks appropriately (SI-2), with other studies also showing the influence of Twitter (e.g., Gough et al. 2017; Guidry et al. 2017), as users can be influenced through extensive online interpersonal conversations (Neubaum and Krämer 2017). Mainstream exposure (e.g., advertorials) also changed intentions to adopt public health advisories due to the mediation of associated emotions (e.g., fear, anxiety) and risk perception (Liu et al. 2022).

However, for many of our communication messaging conditions, the type and source of media show no significant differences in their ability to influence intentions (e.g., recycling surgical masks and washing cloth masks; Table 2). This may raise concerns over the dangers of fake news. By not showing increased trust in potentially more reliable (e.g., local government is considered a trusted source, Lee and Li 2021) or the arguably better regulated mass media versus social media (Salaudeen and Onyechi 2020), people may be open to being influenced by misinformation (Vosoughi et al. 2018). Therefore, to increase sustainability-related intentions, decision-makers and practitioners should be encouraging multiple sources to deliver sustainability information and to do so using a variety of different types of media. However, to help ensure this messaging is successful, decision-makers and practitioners should take measures to mitigate against the spread of misinformation. Such measures may be technological (e.g., making use of platform-based detection curtailing bots to exclude misinformation messages, Lazer et al. 2018). But, since the spread of misinformation is derived from human behaviors, alternative approaches could include communications to dissuade people from spreading misinformation (Vosoughi et al. 2018; Pennycook and Rand 2021).

As with all research, our study has a number of limitations that must be considered when drawing inferences from the results. Broadly, our limitations can be summarized as: (i) potential sampling bias, (ii) whether behavioral intentions lead to changes in behavior, and limitations in (iii) media sources, (iv) media types, and (v) crises. While 18,805 is a large sample, particularly during an ongoing crisis when people, understandably, have many worries other than responding to scientific surveys (e.g., home schooling; Benzeval et al. 2020), it may not be representative of the wider UK population or other countries. Previous research (e.g., Kim and Tandoc Jr 2022; Liu et al. 2022) showed sampling biases, when participants were likely to have more interest in helping tackle COVID-19 because their research was undertaken during the pandemic. Such interests may increase participants’ compliance with guidelines to cope with the COVID-19 pandemic. Thus, the observations of behavioral intentions taken during the COVID-19 pandemic may be biased toward compliance. However, our research was conducted in September 2022 when the COVID-19 virus was substantially less fatal than it was in 2020 (Charumilind et al. 2022), and this suggests that our findings of the effect of media messaging on sustainable intentions is unlikely due to sampling bias (i.e., with an increase in sustainable intentions found within non-mask wearers; Fig. 2). On the other hand, our findings may not be reflective of the effect of media messaging in the height of the pandemic when people may have been more concerned about their health protection rather than pro-environmental practices. In that circumstance, our messaging may have had less impact on the behavioral intention of sustainable practice during the COVID-19 pandemic. Similarly, our findings may be limited to the COVID-19 crisis and may not be replicated for other crises (e.g., the cost-of-living crisis, political uncertainties), opening an avenue for further research. The results presented here may provide preliminary evidence that communications may have a positive impact on sustainable intentions, but each crisis is unique and complex—so such evidence should be viewed with caution. Future research should be conducted to investigate the impact of communication messages on sustainable intentions during other crises, before a meta-analysis is able to draw these results together to make more robust conclusions that are transferable to generalized crises.

Another limitation regarding sampling strategy is related to the use of non-random sampling. This research draws on a judgment sample from Pick My Postcode, with the aim of obtaining a representative sample. The choice of non-probability sampling is considered appropriate when first testing relationships and building theories (Thietart et al. 2007), but may nevertheless pose a threat to the generalizability of the findings. One of the keys to assessing external validity is to evaluate as to whether the sample findings hold consistent across different populations, settings or times (Cook et al. 1979; Thietart et al. 2007). Our participants were drawn from the Pick My Postcode platform, this sample (*N* = 18,805, *M*_age_ = 53.43, 63% female; SI-2) is representative of the general UK population, mapping closely to population density (SI-2-1), but application outside the UK may be limited. Our communication messages might be applicable to the context of EU countries as people from the UK and EU countries have been highly aware of the dramatic impact of the climate crisis and experienced continuously increasing temperatures (McKie 2023), water shortages (Henley et al. 2023), and thus they are more likely willing to adopt sustainable lifestyles than people from other regions (Am et al. 2022; Cromwell and Perkins 2022).

A further limitation of this research is that, while our survey showed significant differences in behavioral intentions, this may not have transferred to actual changes in behaviors (i.e., adoption of more sustainable practices). The intention—behavior gap is large—only about half the time do intentions become actualized (Sheeran and Webb 2016). However, intentions are considered as one of the best predictors of actual behavior (Ajzen 2002). For example, Tarkiainen and Sundqvist (2005) identified that intention can explain up to 83% of the variation of self-reported behavior of sustainable food consumption.

Our research was unable to encompass all possible media sources or media types. Parties other than the ones investigated here have played important roles in public health communications; for example: educational institutions (e.g., in reducing mental health problems during COVID-19, Gao et al. 2020), workplaces (e.g., reducing anxiety during COVID-19, Kay et al. 2022) and medical spokespersons (e.g., social distancing practice, Abu-Akel et al. 2021). Thus, while we found messaging from local government to be particularly effective, future research should contrast this against other potential sources, which may prove more influential. Similarly, our research did not examine the effect of other types of media beyond tweets, advertorials and web-based press. For example, we did not study any mobile-based communication means (e.g., SMS, WhatApps, health applications, etc.). The use of mobile-based health applications has been tested in providing updates of health practices during the outbreak (Srivastav et al. 2021). As above, future studies are encouraged to expand our investigation by examining additional potential media sources (e.g., educational institutions, medical spokespersons, workplaces) and other media types (e.g., mobile-based platform).

## Supplementary Information

Below is the link to the electronic supplementary material.

Supplementary file1 (DOCX 48250 KB)

## Data Availability

The data are freely available via the UK Data Service: “Nguyen, Thi Hong Ngoc and Hassan, Louise and Willcock, Simon (2023). *UK Mask Wearing Behaviour and Attitudes in the COVID-19 Pandemic, Survey 2, 2022.* [Data Collection]. Colchester, Essex: UK Data Service.
