# Supplementary material for: Communications enhance sustainable intentions despite other ongoing crises
Source: Sustain Sci. 2024 Sep 14;19(6):1997–2012. doi: 10.1007/s11625-024-01556-9 (PMC11543749; doi:10.1007/s11625-024-01556-9)
Supplement: Supplementary file 1 — Supplementary file1 (DOCX 48250 KB) [file 11625_2024_1556_MOESM1_ESM.docx]

SUPPLEMENTARY INFORMATION

# SI-1: The survey experiment conducted in this study

## Picking up face mask litter

How likely are you to pick up face covering litter you see in public places? (1-*Extremely unlikely* to 7-*Extremely likely*)

Why are you unlikely to pick up face covering litter you see in public places? Tick all that apply.

□ Because I don't think that it is safe

□ Because I don't see any face covering litter

□ Because I don't pick up other types of litter anyway

□ Because it is too much effort

□ Because I am not interested in picking up face mask litter

□ Because I don’t think it is my responsibility to pick up others’ face mask litter

□ Because people that I know do not pick up face mask litter

□ Other (please specify)

[Communication message presented]

Based on this information, how likely are you to take part in your local face covering litter pick up? (1-*Extremely unlikely* to 7-*Extremely likely*)

Why are you unlikely to pick up face covering litter you see in public places? Tick all that apply.

□ Because I still don't feel that it is safe

□ Because I don't see any face covering litter

□ Because I don't pick up other types of litter anyway

□ Because it is too much effort

□ Because I am not interested in picking up face mask litter

□ Because I don’t think it is my responsibility to pick up others’ face mask litter

□ Because people that I know do not pick up face mask litter

□ Because I don’t trust the information presented to me

□ Because the information presented me is not relevant

□ Because the information presented to me is not persuasive

□ Because of health-related reasons

□ Because I am not available on the 10th of September

□ Other (please specify)

## Recycling surgical masks

Do you currently recycle your surgical face covering?

| ⃝ Almost always | ⃝ No | ⃝ Sometimes |
| --- | --- | --- |

[Communication message presented]

Based on this information, how likely are you to recycle your surgical face covering? (1-*Extremely unlikely* to 7-*Extremely likely*)

Why are you unlikely to employ these techniques when disposing of your surgical face coverings? Tick all that may apply.

□ Because there is not a mask recycling point/bin near me

□ Because I cannot afford to purchase a recycling box from TerraCycle or Reworked

□ Because it is too much effort

□ Because I don't trust the information presented to me

□ Because the information presented me is not relevant

□ Because the information presented to me is not persuasive

□ Because people that I know do not employ these techniques when disposing of their surgical face coverings

□ Other (please specify)

## Disinfecting FFP masks

Please indicate how many times you wear your FFP face covering for before disposing of it

| ⃝ Only once | ⃝ 2-3 times | ⃝ 4-6 times | ⃝ 7-9 times | ⃝ > 10 times |
| --- | --- | --- | --- | --- |

Do you take measures to disinfect your FFP type face covering in-between wearing?

| ⃝ Yes | ⃝ No | ⃝ I only wear FFP type face coverings once |
| --- | --- | --- |

[Communication message presented]

Given this information about disinfecting your face covering, please indicate how many times you will wear your FFP type face covering in future before disposing of it

| ⃝ Only once | ⃝ 2-3 times | ⃝ 4-6 times | ⃝ 7-9 times | ⃝ > 10 times |
| --- | --- | --- | --- | --- |

How likely are you to use this method to disinfect FFP type face coverings in the future? (1-*Extremely unlikely* to 7-*Extremely likely*)

Why are you unlikely to employ these practices to disinfect and reuse your FFP face covering? Tick all that apply.

□ I don't trust that this will effectively disinfect my face coverings

□ I don't think the information presented to me is relevant

□ I don't think the information presented to me is persuasive

□ I don't want to buy this many face coverings

□ I don't use this type of face covering regularly enough

□ The methods seem too difficult

□ Because people that I know do not employ these practices to disinfect and reuse their FFP face coverings

□ Other (please specify)

## Washing cloth face coverings

Generally, do you wash your cloth face covering...

| ⃝ Separately from the rest of your household washing | ⃝ With your normal household washing | ⃝ By hand |
| --- | --- | --- |

What temperature do you wash your cloth face covering at?

| ⃝ 30°C | ⃝ 40°C | ⃝ 50°C | ⃝ 60°C | ⃝ 70°C |
| --- | --- | --- | --- | --- |
| ⃝ 80°C | ⃝ 90°C | ⃝ Other (please specify) | ⃝ I don't know |  |

[Communication message presented]

Based on this information, what temperature will you wash your cloth face covering at?

| ⃝ 30°C | ⃝ 40°C | ⃝ 50°C | ⃝ 60°C | ⃝ 70°C |
| --- | --- | --- | --- | --- |
| ⃝ 80°C | ⃝ 90°C | ⃝ Other (please specify) | ⃝ I don't know |  |

Why would you not wash your cloth face covering at 60ºC or above?

□ Because it is too much effort

□ Because I don't trust that washing at the recommended temperature kills any coronavirus and makes the face mask safe to use again

□ Because the information presented me is not relevant

□ Because the information presented to me is not persuasive

□ Because people that I know do not wash their cloth face covering at the recommended temperature

□ Other (please specify)

□ I do not do any washing at 60ºC

# SI-2: Supplementary results


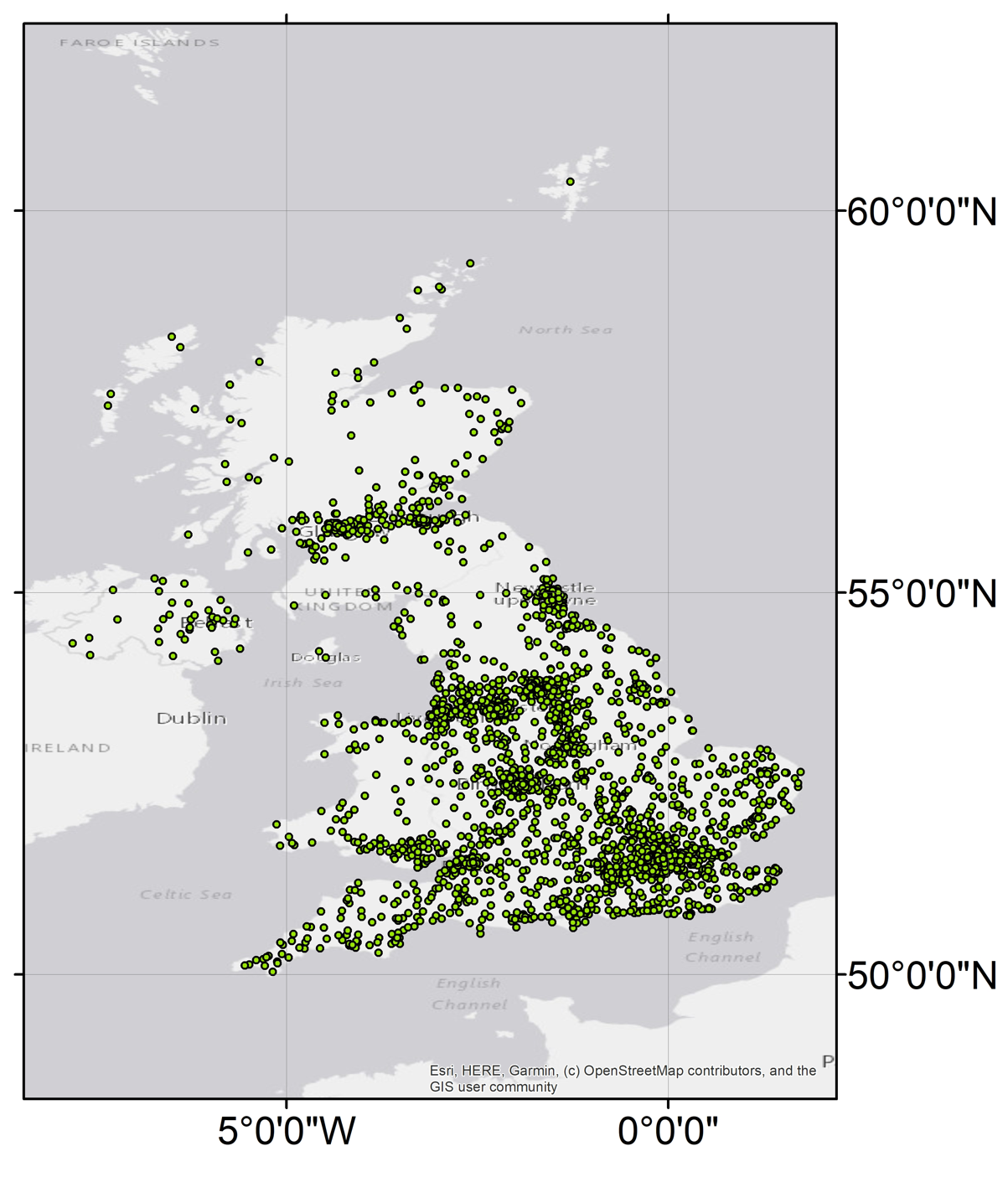
SI-2-1: Additional descriptive statistics

**Figure S1:** Population distribution of our 18,805 participants within the UK, each respondent represented by a green dot on the map


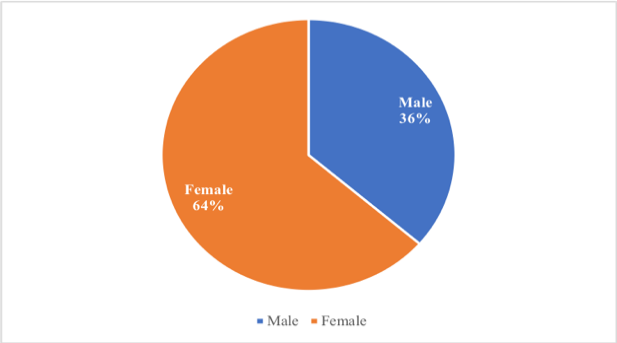

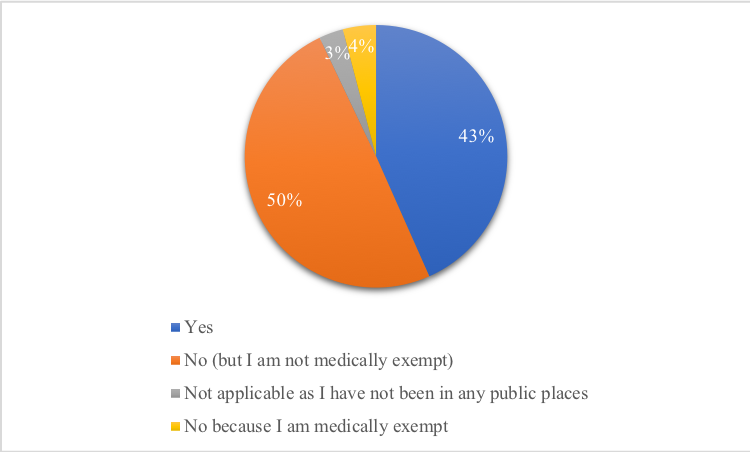

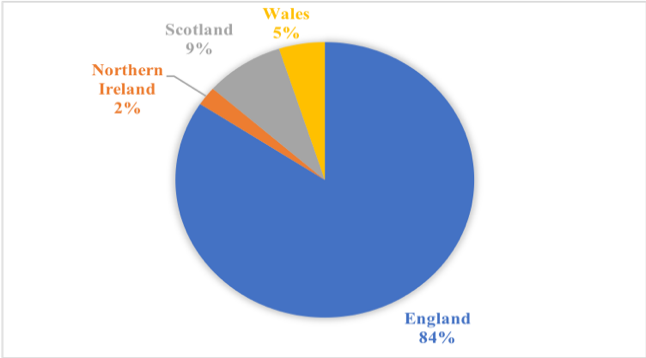


**Figure S2:** Summary description of our 18,805 participants, showing a) gender ratio, b) location within the UK, and c) mask-wearing behaviors.


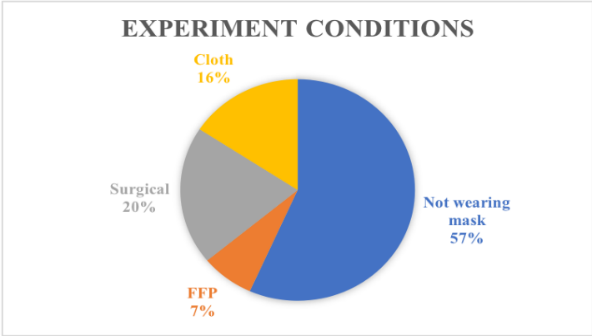

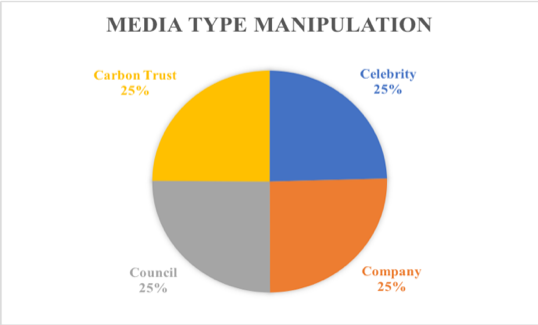

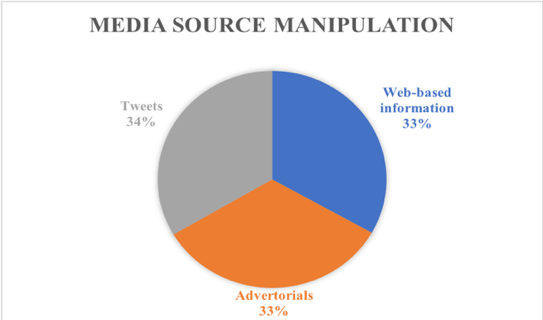


**Figure S3:** Summary description of how 18,805 participants were divided amongst our experimental conditions, showing a) mask-wearing behavior, b) media type, and c) media source

**Figure S4**: Likelihood of picking up mask litter before versus after messaging from different media types and sources, ranging from 1- *Extremely unlikely* to 7-*Extremely likely*.


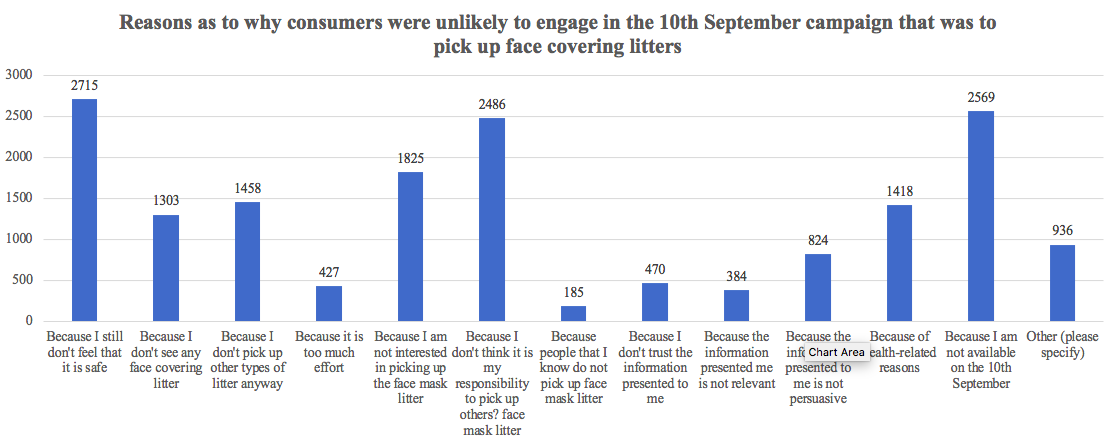


**Figure S5**: Frequency of reasons why participants were unlikely to engage in the campaign to pick up face covering litter


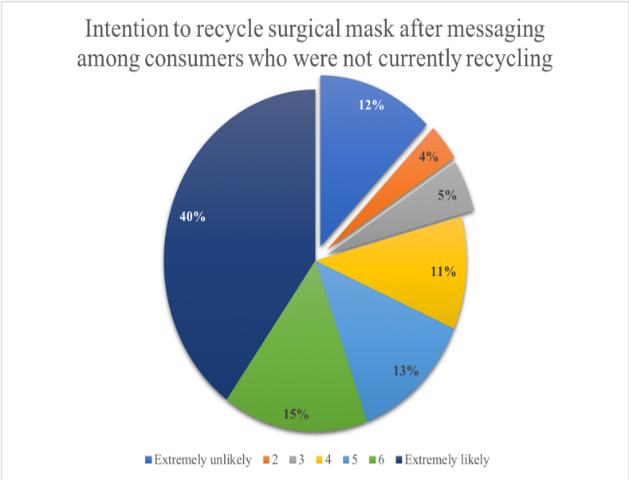

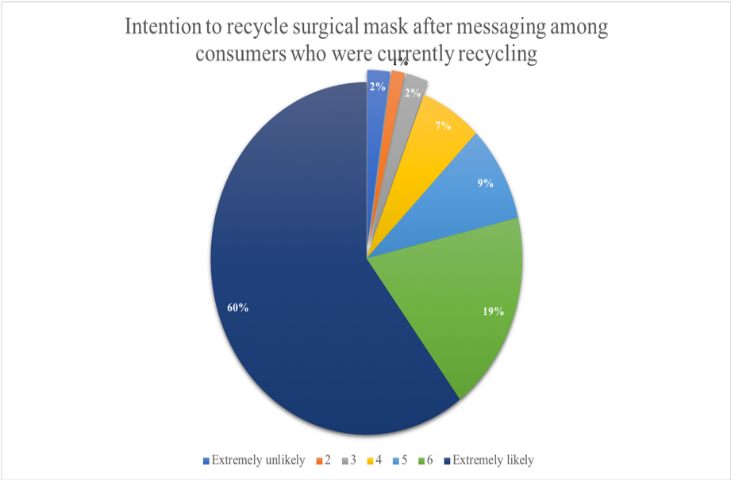


**Figure S6**: Intention to recycle surgical masks after the communication message was presented both a) for those currently recycling masks, and b) those who were not currently recycling masks


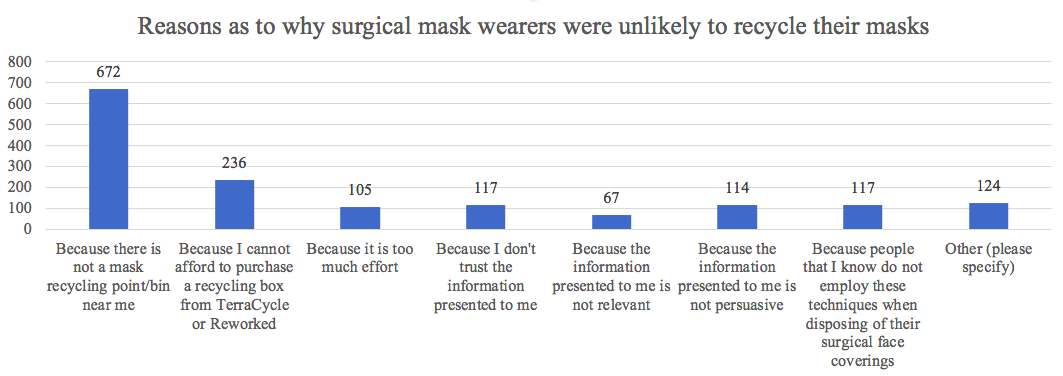


**Figure S7:** Frequency of reasons why surgical mask wearers were unlikely to recycle their masks


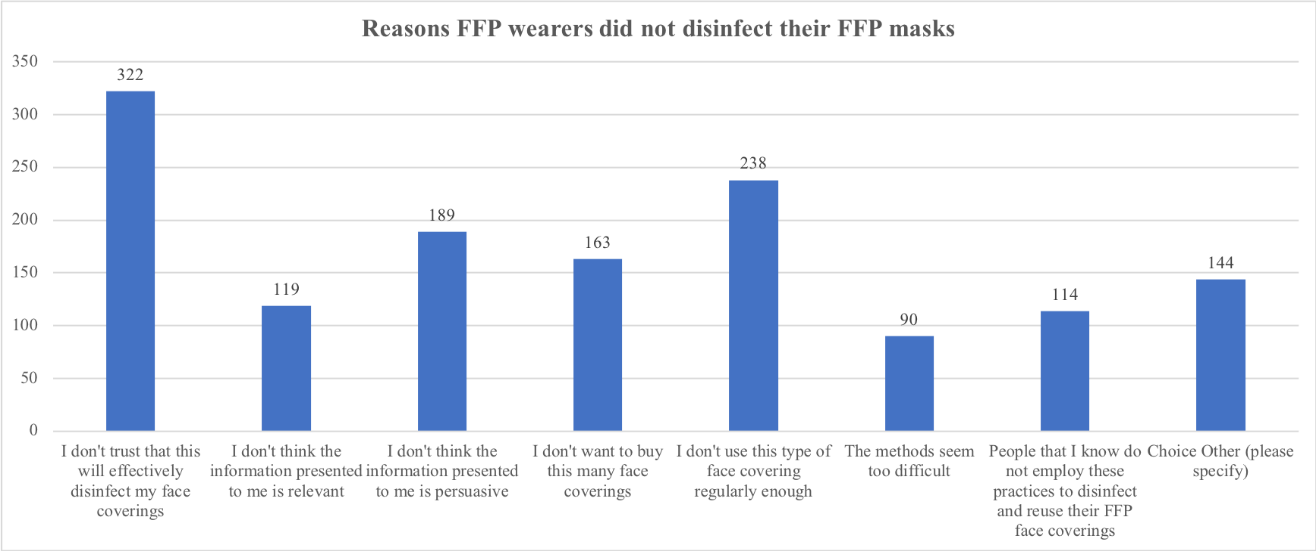


**Figure S9:** Frequency of reasons for reusing FFP mask fewer times than was recommended in our communication


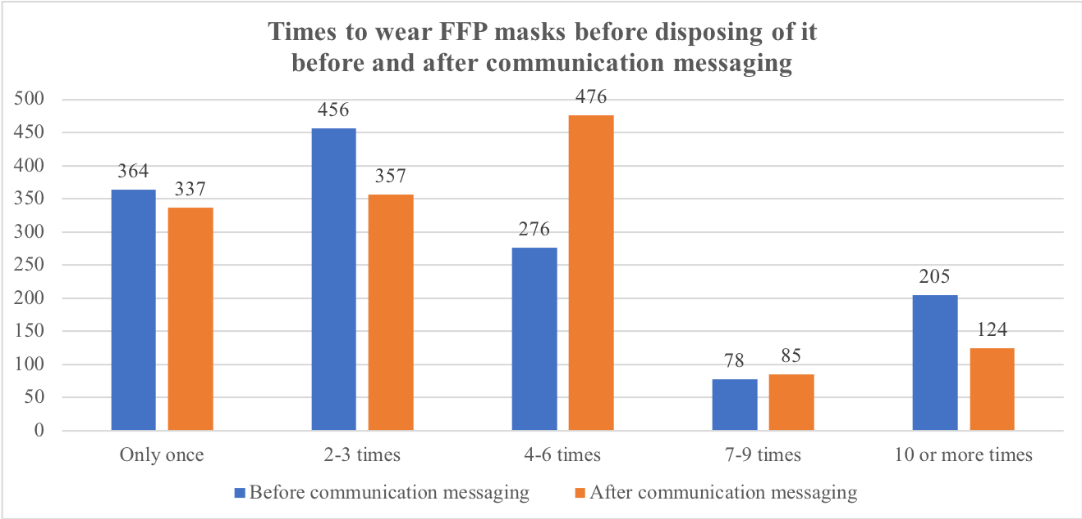


**Figure S8**: Frequencies that people reported to reuse their FFP masks before (blue) versus their intentions after (orange) our information was presented


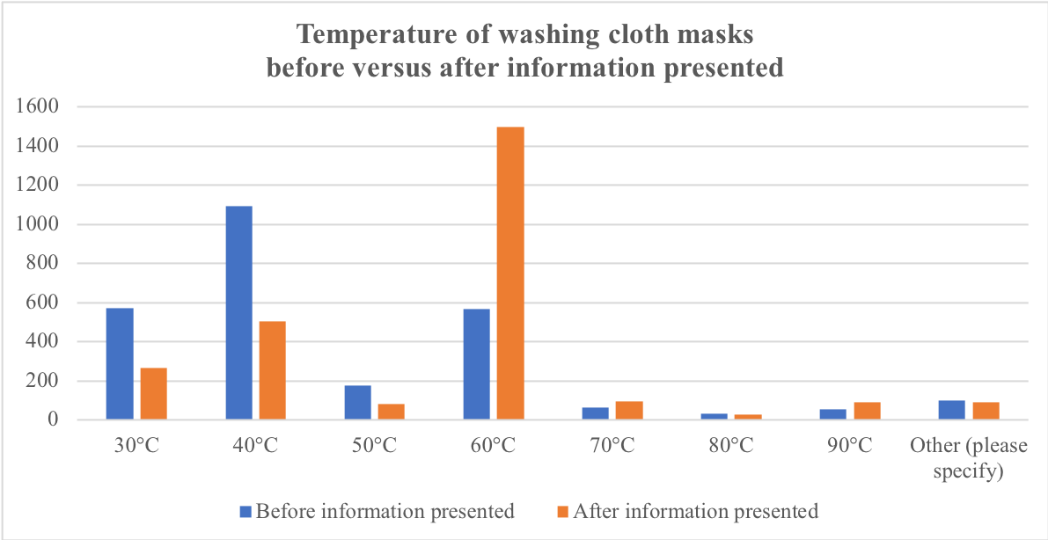


**Figure S10:** Frequency of temperature of washing cloth masks before (blue) versus intentions after (orange) our information was presented


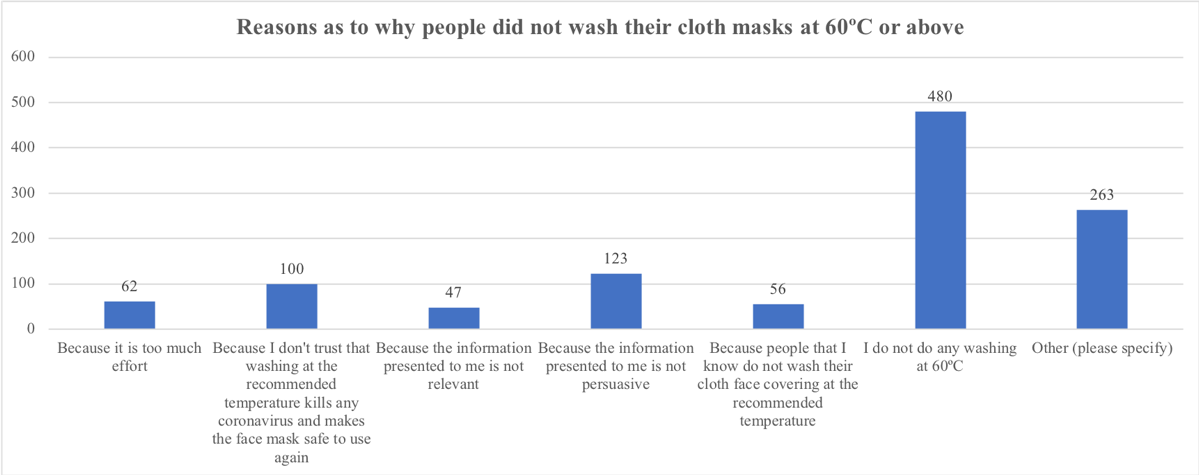


**Figure S11**: Frequencies of reasons for not washing their cloth masks at 60ºC or above as recommended in our messaging

SI-2-2: Additional statistical analysis and results

## Picking up face masks

As indicated in the *Results* section of the main text, intentions to pick up mask litter was significantly different between before and after the advisory was presented (F(1,10638)=15.01, *p*<.001) and this change was statistically significant between groups of media sources (F(3,10638)=11.00, *p*<.001). Specifically, messages from local government greatly improved the likelihood of picking up litter, greater than any changes determined by messaging from other sources of media (celebrity, company, NGO; Figure S11). This change by local government’s communication was shown across different types of media such as Tweets (M_difference_=1.12, M_before_=2.16, M_after_=3.28), web-based information (M_difference_=0.84, M_before_=2.28, M_after_=3.12), and advertorial (M_difference_=0.85, M_before_=2.40, M_after_=3.25). The Sankey diagram also supports this finding that more people rated high intentions to pick up face masks (at the midpoint of the scale (4) or above) after viewing messages from the local government (N=1250, 46.21%) than any other sources such as NGO (N=1061, 40.57%), company (N=1050, 38.56%) and celebrity (N=1030, 39.48%; Figure S12).


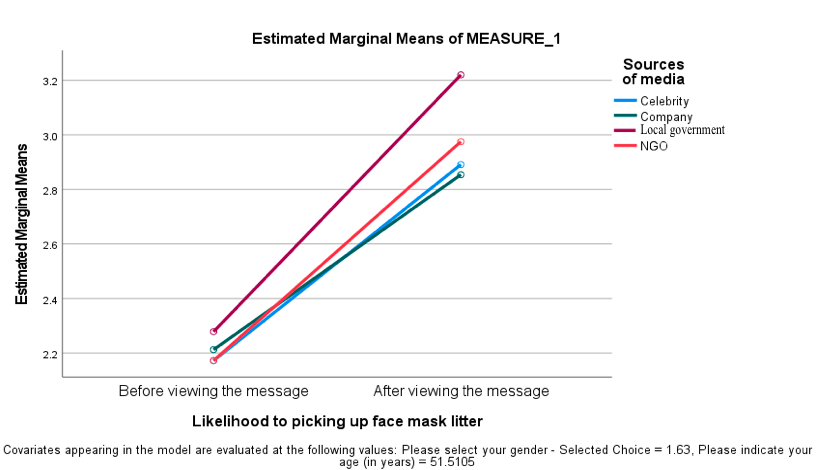


**Figure S12**: Changes in the likelihood to pick up face mask litter before and after viewing the message across four media sources

**Figure S13:** Sankey diagram demonstrating flows of changes in consumers’ intention to pick up face mask litter across multiple sources of media

Note: Participants were asked to indicate the degree to which they were likely to recycle their surgical mask on a seven-point scale (from 1-*Extremely unlikely* to 7-*Extremely likely*) before (Pre_) viewing the messaging and after (Post_) viewing the messaging


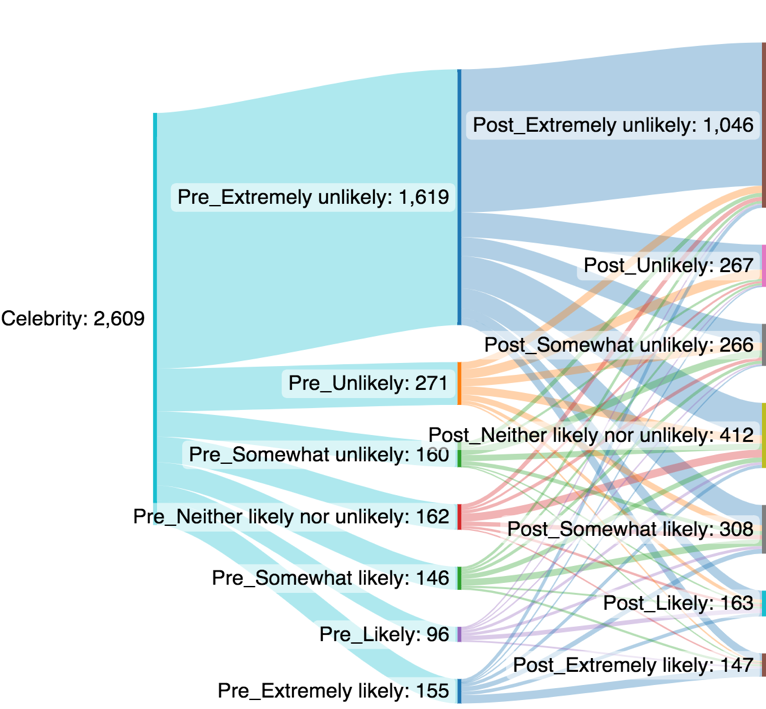

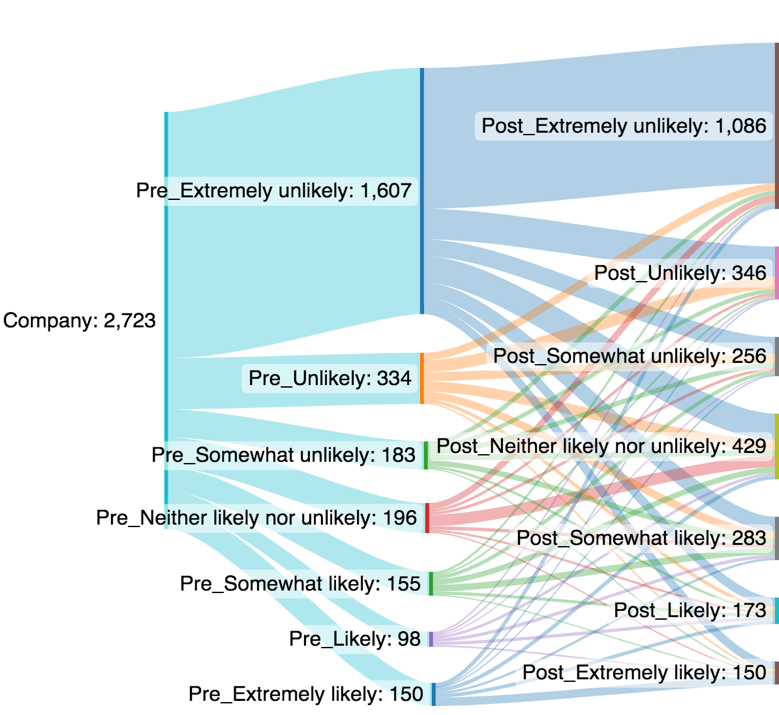

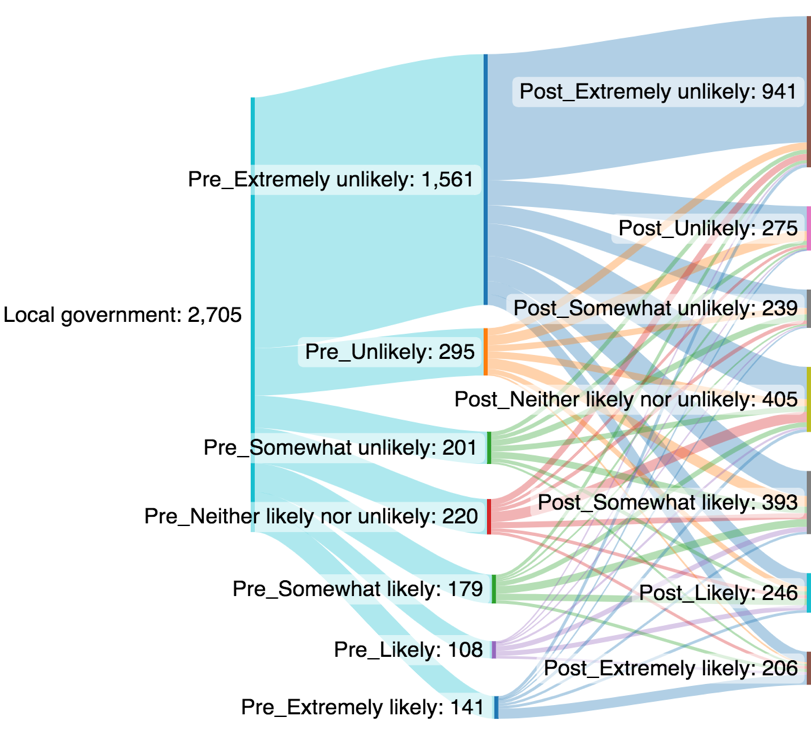

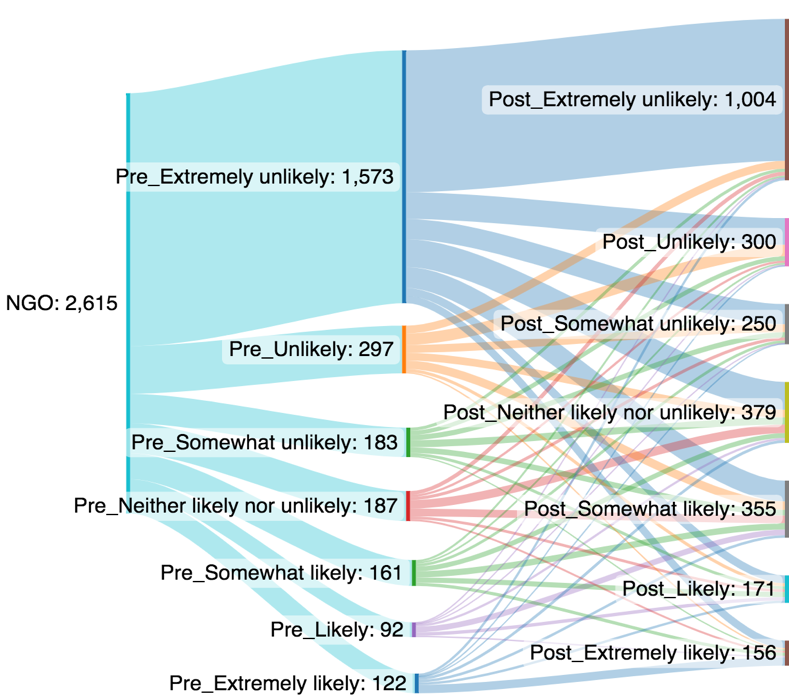


## Disinfecting FFP masks

The interaction of media types and initial report significantly influenced the intention of the number of times an FFP mask would be worn following the communication messaging (b= -0.16, *p*=.03). The frequencies that people disinfected their FFP masks were coded from 1 to 5 for ‘*Only once*’, ‘*2-3 times*’, ‘*4-6 times*’, ‘*7-9 times*’, and *‘10 or more times*’ respectively. For those who reported previously reusing masks from 1 to 3 times, Tweets was a the most effective to increase their disinfection times (M range: 1.64 to 2.39, *p*<.001), followed by advertorials (M range: 1.63 to 2.07, *p*<.001), and web-based information (M range: 1.45 to 2.23, *p*<.001; Figure S13). Meanwhile, those who disinfected their FFP masks 7-9 times were more likely to decrease to 4-6 times by viewing the message via advertorials (M=3.21, *p*<.001), followed by tweets (M=3.52, p<.001) and web-based information (M=3.56, *p*<.001; Figure S13). Compared to other media types, most people disinfected their FFP masks from 4 to 6 times after viewing the messages from tweets (N=156, 36.03%; Figure S14). Fewest people disinfected FFP masks from 7 to 9 times after viewing the messages from advertorials (N=23, 4.83%; Figure S14). Those who previously used FFP 4-6 times did not change the frequency of disinfecting their FFP masks after reading the information delivered from any media types (M_Web_=2.90, M_Advertorials_=2.69, M_Tweets_=2.81; Figure S14).


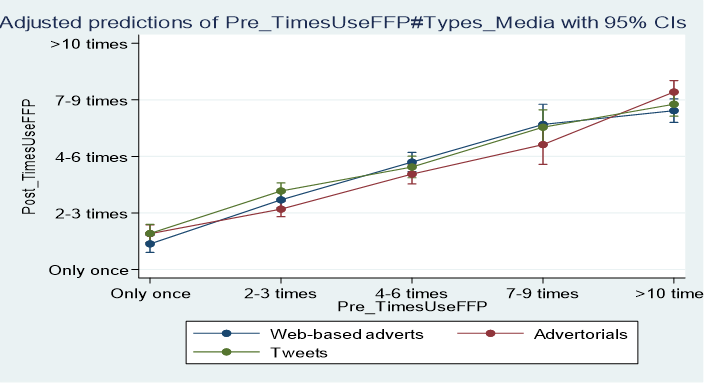

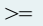

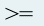

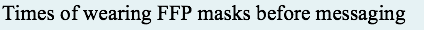

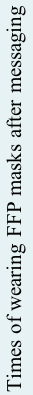


**Figure S14:** Plotting the interaction effect of media types and initial report predicting the frequencies of using FFP masks after viewing the message.


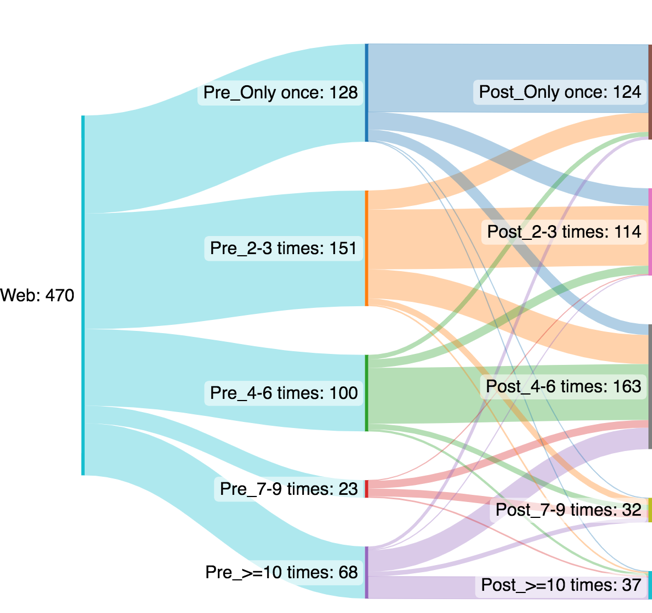

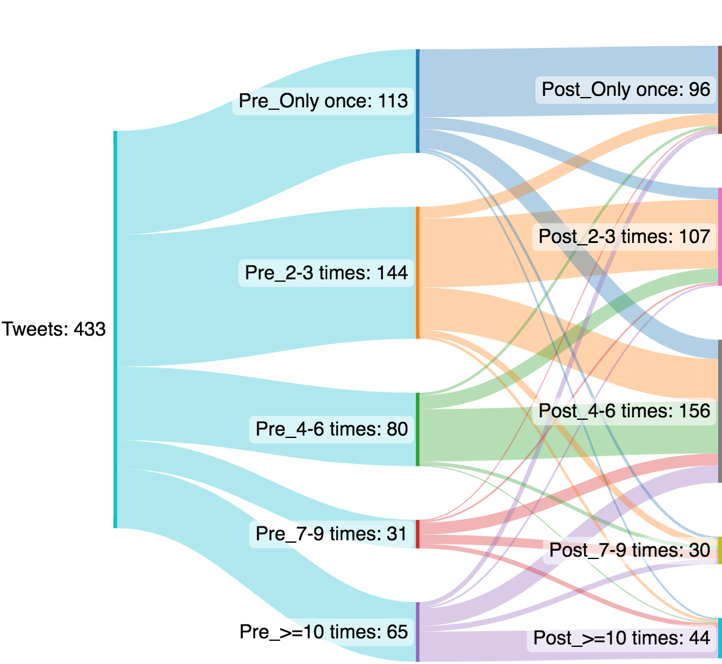

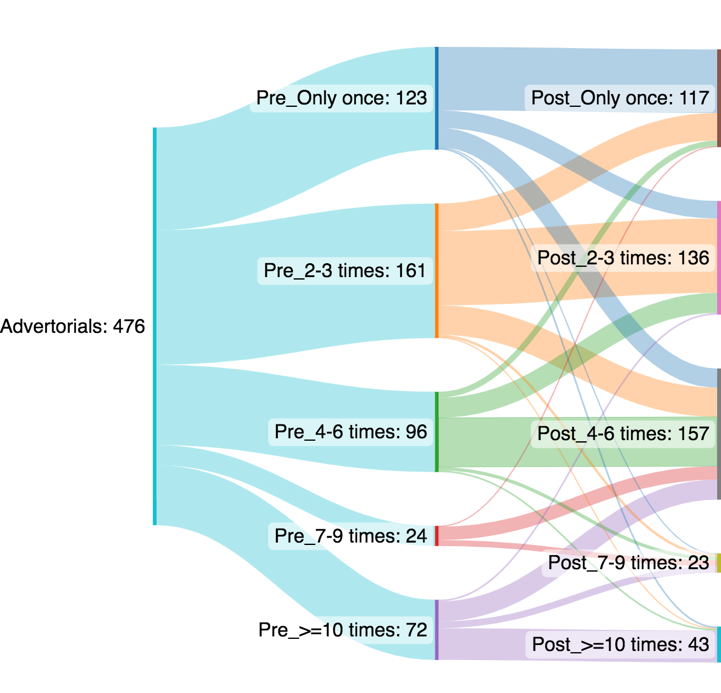


**Figure S15:** Sankey diagram demonstrating flows of changing in consumers’ intention to adopt FFP disinfection measures across multiple types of media

*Note*: Participants were asked to report their times of disinfecting their FFP masks before (Pre_) viewing the messaging and the times that they would disinfect their FFP masks after (Post_) viewing the messaging (i.e., *Once; 2-3 times; 4-6 times; 7-9 times; 10 or more times*)

**Table S1:** PROBIT model predicting the frequency that consumers disinfected their FFP masks after the message was presented

| Predictors | Coef. | SE | z | P>\|z\| |
| --- | --- | --- | --- | --- |
| Gender | 0.11 | 0.06 | 1.94 | 0.05 |
| Age | 0.006 | 0.002 | 2.57 | 0.01 |
| Maximum times of wearing FFP masks before messaging | 1.05 | 0.16 | 6.71 | <0.001 |
| Types_Media | 0.35 | 0.20 | 1.72 | 0.09 |
| Sources_Media | 0.21 | 0.16 | 1.36 | 0.17 |
| Maximum times of wearing FFP masks before messaging x Types_Media | -0.16 | 0.07 | -2.19 | 0.03 |
| Maximum times of wearing FFP masks before messaging x Sources_Media | -0.1 | 0.05 | -1.80 | 0.07 |
| Sources_Media x Types_Media | -0.08 | 0.07 | -1.04 | 0.30 |
| Maximum times of wearing FFP masks before messaging x Sources_Media x Types_Media | 0.05 | 0.03 | 1.86 | 0.06 |

**Table S2**: PROBIT regression predicting consumers' choice of temperature to wash their cloth masks after the message was presented

| Predictors | Coef. | SE | z | P>\|z\| |
| --- | --- | --- | --- | --- |
| Gender | -0.06 | 0.05 | -1.36 | .17 |
| Age | 0.003 | 0.001 | 1.97 | 0.05 |
| Choices of temperature washing cloth mask before messaging | 0.40 | 0.09 | 4.69 | <0.001 |
| Types_Media | -0.07 | 0.12 | -0.59 | 0.55 |
| Sources_Media | -0.09 | 0.10 | -0.94 | 0.35 |
| Choices of temperature washing cloth mask before messaging x Types_Media | -0.005 | 0.04 | -0.13 | 0.90 |
| Choices of temperature washing cloth mask before messaging x Sources_Media | 0.002 | 0.03 | 0.07 | 0.94 |
| Sources_Media x Types_Media | 0.04 | 0.05 | 0.78 | 0.43 |
| Choices of temperature washing cloth mask before messaging x Sources_Media x Types_Media | 0.004 | 0.01 | 0.25 | 0.80 |

**Table S3: [More details of Table 2]** A summary of the impact of each communication messaging condition on sustainability-related intentions of our 18,805 participants, highlighting the overall outcome of the messaging intervention (i.e., captured by the adopted intention), the magnitude and significance of the change compared to stated intentions prior to the messaging intervention, as well as any significant interaction terms.

| Communication messaging condition | Overall outcome of the messaging intervention (i.e., captured by the adopted intention) | Overall change (i.e., before vs after communication messaging) | Effect of past behavior on the overall change | Effect of media types * past behavior on the overall change | Effect of media sources * past behavior on the overall change | Effect of media types * media sources* past behavior on the overall change |
| --- | --- | --- | --- | --- | --- | --- |
| Picking up face mask litter | A one sample t-Test found that people were not likely to pick up face mask litter either before or after the communication (significantly lower than the midpoint of the scale (4), M_before_=2.21, t=-100.56, df=10651, p<.001; M_after_=2.99, t=-52.81, df=10651, *p*<.001), | Repeated measure ANOVA: F(1,10638)=15.01, *p*<.001  A paired-sample *t*-Test found that intention to pick up mask litter was improved after viewing the message : (M_before_=2.21, M_after_=2.99, M_difference_=0.78, df=10651, *p*<.001) |  | Repeated measure ANOVA: No interaction effect found | Repeated measure ANOVA: F(3,10638)=11.00, *p*<.001 | Repeated measure ANOVA: No interaction effect found |
| Recycling surgical masks | A one-sample *t*-Test analysis showed the likelihood that these consumers recycled their surgical masks after the communication messaging were significantly higher than the midpoint of the scale (4) (1-*extremely unlikely* to 7-*extremely likely*) (M=5.61, t=53.09, df=3797, *p*<.001) | Number of people previously reported NOT recycling (N=2050,54%), SOMETIMES (N=638, 16.8%), ALWAYS (N=110, 29.2%) categories are compared with number of people, after viewing the message, reported intention at the midpoint of the 7-point scale (4) or above (N=3266, 86%; in which 1874 people (49.3%) reported ‘*Extremely likely*’) | Univariate ANOVA: F(2,3760)=157.29, *p*<.001.  A one-sample *t*-Test found that those reported NO previously rated significantly higher likelihood than mid-scale (4) (N=2050, M=5.15, df=2049, *p*<.001); same for those reported SOMETIMES: (N=638, M=5.84, df=637, *p*<.001); and reported ALWAYS: (N=1110, M=6.32, df=1109, *p*<.001). | Univariate ANOVA: No interaction effect found | Univariate ANOVA: No interaction effect found | Univariate ANOVA: No interaction effect found |
| Disinfecting FFP masks | More people intended to disinfect their FFP 4-6 times after viewing the message (N=476, 34.5%), followed by 2-3 times (N=357, 25.9%) and once (N=337, 24.4%) | Numbers of people choose to disinfect FFP 4-6 times before (N=276, 20%) is much less than after (N=476, 34.5%) | PROBIT regression: b=1.05, z=6.71, *p*<.001 | PROBIT regression: b=-0.16, z=-2.19, *p*=.03. | PROBIT regression: No interaction effect found | PROBIT regression: No interaction effect found |
| Washing cloth face coverings | Majority of people chose to wash their cloth masks at 60ºC (N=1499, 56.50%) after viewing the message, followed by below 60ºC (N=851, 32.08%) and above 60ºC (N=215, 8.10%) | Majority of the sample reported washing at 40ºC (N=1091, 41.12%) prior to the advice provided. People chose to wash their cloth masks at 60ºC increased from 565 (21.30%) to 1499 (56.50%) after viewing the message | PROBIT regression: b=0.40, z=4.69, *p*<.001 | PROBIT regression: No interaction effect found | PROBIT regression: No interaction effect found | PROBIT regression: No interaction effect found |
